# Statistics of Breathing

**Published:** 1890-03

**Authors:** 


### Statistics of Breathing.

In each respiration an adult inhales one pint of air.

A man respires sixteen to twenty times a minute, or twenty thousand times a day; a child twenty-five to thirty-five times a minute.

While standing, the adult respiration is twenty-two; while lying, thirteen.

The superficial surface of the lungs, *i. e.*, of their alveolar space, is two hundred square yards.

The amount of inspired air in twenty-four hours is ten thousand litres (about ten thousand quarts).

The amount of oxygen absorbed in twenty-four hours is five hundred litres (744 grammes); and the amount of carbonic acid gas expired in the same time, four hundred litres (911.5 grammes).

Two-thirds of the oxygen absorbed in twenty-four hours is absorbed during the night hours from 6 p. m. to 6 a. m.

Three-fifths of the total carbonic acid is thrown off in the day time.

The pulmonary surface gives off one hundred and fifty grammes of water daily in the state of vapor.

An adult must have at least three hundred and sixty litres of air an hour.

The heart sends through the lungs eight hundred litres of blood hourly, and twenty thousand litres, or five thousand gallons, daily. The duration of inspiration is five twelfths, of expiration, seven-twelfths, of the whole respiratory act, but during sleep inspiration occupies ten-twelfths of the respiratory period.—*Annals of Hygiene.*

---

Senator Ingalls has introduced a bill into the Senate establishing a Board of Medical Examiners for the District of Columbia. The bill provides that the Board shall consist of ten physicians or surgeons, three dental surgeons, and, in addition, five homœopathic practitioners of medicine. The term of office is to be four years.

